# A case report: right upper lobectomy with middle lobe preservation after right lower lobectomy

**DOI:** 10.1186/s40792-015-0026-4

**Published:** 2015-02-19

**Authors:** Hitoshi Igai, Mitsuhiro Kamiyoshihara, Natsuko Kawatani, Takashi Ibe, Kimihiro Shimizu

**Affiliations:** Department of General Thoracic Surgery, Maebashi Red Cross Hospital, 3-21-36 Asahi-cho, Maebashi, Gunma 371-0014 Japan

**Keywords:** Right upper lobectomy, Middle lobe preservation, Right lower lobectomy, Pulmonary metastasis

## Abstract

Few reports have described right upper and lower lobectomy with preservation of the middle lobe because of the risk of middle lobe torsion or emphysematous change. Herein we describe a successful result following lobectomy with preservation of the middle lobe for metachronous pulmonary metastasis originating from colon cancer in the right upper lobe after initial right lower lobectomy. A 69-year-old man who had undergone right lower lobectomy for pulmonary metastasis originating from colon cancer 3 years earlier was diagnosed as having suspected metachronous pulmonary metastasis in the right upper lobe. Because preoperative computed tomography (CT) indicated that the distance between the tumor and the entrance of the upper bronchus was 20 mm, it was considered difficult to achieve complete resection by a wedge resection or segmentectomy. Furthermore, preoperative CT demonstrated compensatory hypertrophy of the middle lobe and elevation of the right diaphragm, thus reducing the size of the thorax. Therefore, right upper lobectomy with middle lobe preservation was planned. The operation was performed using a totally thoracoscopic approach. Adhesion of the upper lobe to the chest wall was easily detached. As the middle lobe adhered to the chest wall, this served to prevent middle lobe torsion. The fissure between the upper and middle lobes had fused because of adhesion resulting from the initial lower lobectomy. Therefore, an ‘anterior fissureless approach’ was adopted to avoid any postoperative air leakage. There were no intraoperative problems, and the postoperative course was uneventful. The patient was discharged on postoperative day 6. Pathological examination of the specimen confirmed that the tumor was a metachronous pulmonary metastasis originating from the colon cancer. Four months after the operation, he had no requirement for additional oxygen support, and postoperative CT demonstrated a sufficiently expanded residual middle lobe without emphysematous change.

## Background

Few reports have described right upper and lower lobectomy with preservation of the middle lobe because of the risk of middle lobe torsion or emphysematous change.

Herein we describe a successful result following lobectomy with preservation of the middle lobe for metachronous pulmonary metastasis originating from the colon cancer in the right upper lobe after initial right lower lobectomy.

## Case presentation

**Figure 1:**
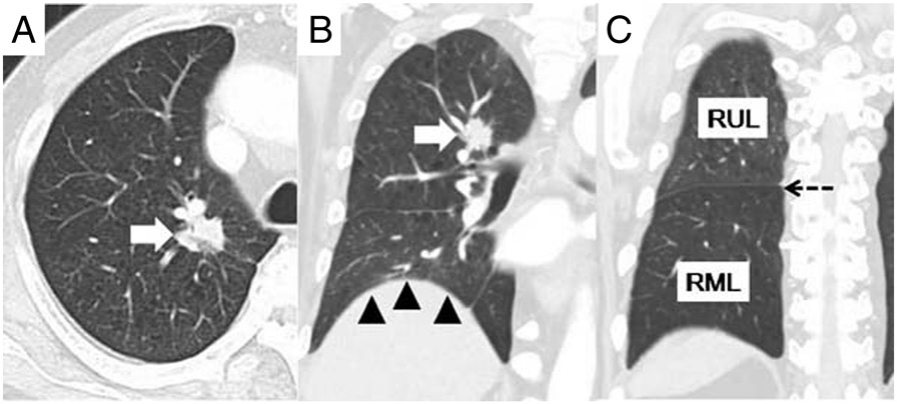
**Initial chest computed tomography. (A)** Preoperative chest computed tomography (CT) demonstrates a 30-mm mass shadow (arrows) in the right upper lobe, and the distance between the tumor and the entrance of the upper bronchus is 20 mm. **(B, C)** Compensatory hypertrophy of the middle lobe and elevation of the right diaphragm (arrowheads), reducing the size of the thorax. Dotted arrow revealed interlobar fissure between right upper and middle lobe. RUL, right upper lobe, RML, right middle lobe.

A 69-year-old man who had undergone right lower lobectomy 3 years earlier for pulmonary metastasis originating from colon cancer consulted our hospital because of an abnormal chest shadow. Initial chest computed tomography (CT) revealed a 30-mm mass shadow in the right upper lobe (Figure 1). Metachronous pulmonary metastasis originating from the colon cancer was suspected. Transbronchiolar lung biopsy was not performed at the patient’s request, and therefore surgical resection was planned.

Preoperative forced vital capacity (FVC) and forced expiratory volume in 1 second (FEV1.0) were 2,580 and 1,330 ml, respectively. Because radiography indicated that the distance between the tumor and the entrance of the upper bronchus was 20 mm, it was considered difficult to achieve complete resection by a wedge resection or segmentectomy. It was anticipated that intrathoracic adhesions resulting from the initial lower lobectomy would prevent postoperative torsion of the residual middle lobe. Furthermore, preoperative CT demonstrated compensatory hypertrophy of the middle lobe and elevation of the right diaphragm, thus reducing the size of the thorax. We expected that this volume matching between the residual middle lobe and the thoracic cavity would prevent emphysematous change in the residual middle lobe in the long term. Therefore, right upper lobectomy with middle lobe preservation was planned. For upper lobectomy, the predicted FEV1.0 was 1,064 ml [1].

The operation was performed using a totally thoracoscopic approach. Adhesion of the upper lobe to the chest wall was easily detached. As the middle lobe adhered to the chest wall, this served to prevent middle lobe torsion. The fissure between the upper and middle lobes had fused because of adhesion resulting from the initial lower lobectomy. Therefore, an ‘anterior fissureless approach’ was adopted to avoid any postoperative air leakage [2]. There were no intraoperative problems, and the postoperative course was uneventful. The patient was discharged on postoperative day 6. Pathological examination of the specimen confirmed that the tumor was a metachronous pulmonary metastasis originating from the colon cancer.

**Figure 2:**
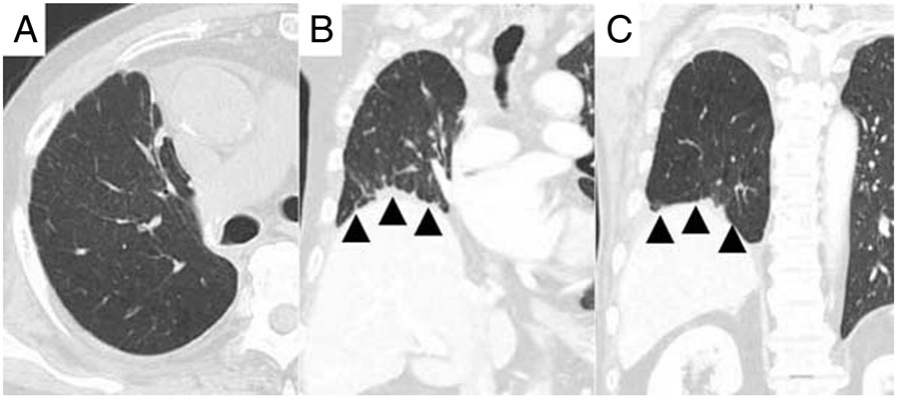
**Postoperative CT of the residual middle lobe. (A,** **B,** **C)** CT 4 months after the operation shows that the residual middle lobe is sufficiently expanded without emphysematous change. There is no evident volume mismatch in the residual middle lobe and thoracic cavity. Arrowheads revealed more elevated right diaphragm compared to preoperation.

After 4 months of follow-up, the patient is alive without any signs of further metachronous metastatic lung cancer and requires no additional oxygen support. Postoperative CT revealed that the residual middle lobe was sufficiently expanded without emphysematous change (Figure 2). There was no volume mismatch in the residual middle lobe and thoracic cavity.

## Conclusions

When right upper lobectomy is required for lung disease after initial right lower lobectomy, pneumonectomy can be considered instead of lobectomy with preservation of the middle lobe. However, a previous study reported that the rates of operative mortality and morbidity in patients undergoing complete pneumonectomy for malignant or benign disease were 10.3% and 55.1%, respectively, and that 7.9% of the patients overall developed bronchopleural fistula [3].

On the other hand, preservation of the middle lobe in the absence of the upper and lower lobes may produce torsion. To prevent such torsion in the present case, the middle lobe adhesions to the chest wall were not removed, and this prevented the residual middle lobe from becoming twisted. In case without such adhesions, Moriyama and colleagues suggested placement of a latissimus dorsi flap through the intercostal space diagonally in the thoracic cavity and fixation of the preserved middle lobe with the flap to prevent postoperative torsion [4].

A further potential problem was the possibility of emphysematous change in the residual middle lobe after surgery due to a volume mismatch in the middle lobe and thoracic cavity. In the present case, however, no such emphysematous changes were observed by follow-up chest CT, because during the 3 years after the initial lower lobectomy, the middle lobe had undergone compensatory hypertrophy and the right diaphragm had become elevated, thus reducing the size of the thoracic cavity. We concluded that these changes over the course of several months or years after initial lobectomy had created favorable conditions for surgical intervention. In previously reported examples of right lower lobectomy with middle lobe preservation after initial right upper lobectomy, 40 and 4 years had passed since the initial lobectomy [5,6].

Accordingly, we consider it important to prevent postoperative torsion by preserving only the middle lobe in the perioperative period, and that a minimal degree of volume mismatching, as confirmed by preoperative CT, should be able to prevent emphysematous changes in the residual middle lobe. In such cases, right upper lobectomy with middle lobe preservation after right lower lobectomy would be a more appropriate surgical procedure than complete pneumonectomy.

## Consent

Written informed consent was obtained from the patient for publication of this case report and any accompanying images. A copy of the written consent is available for review by the Editor-in-Chief of this journal.

## Abbreviations

CT: computed tomography
FVC: forced vital capacity
FEV1.0: forced expiratory volume in 1 second
